# Bayesian Hierarchical Models Combining Different Study Types and Adjusting for Covariate Imbalances: A Simulation Study to Assess Model Performance

**DOI:** 10.1371/journal.pone.0025635

**Published:** 2011-10-10

**Authors:** C. Elizabeth McCarron, Eleanor M. Pullenayegum, Lehana Thabane, Ron Goeree, Jean-Eric Tarride

**Affiliations:** 1 Department of Clinical Epidemiology and Biostatistics, McMaster University, Hamilton, Ontario, Canada; 2 Programs for Assessment of Technology in Health (PATH) Research Institute, St. Joseph's Healthcare Hamilton, Hamilton, Ontario, Canada; 3 Biostatistics Unit, St. Joseph's Healthcare Hamilton, Hamilton, Ontario, Canada; University of Glasgow, United Kingdom

## Abstract

**Background:**

Bayesian hierarchical models have been proposed to combine evidence from different types of study designs. However, when combining evidence from randomised and non-randomised controlled studies, imbalances in patient characteristics between study arms may bias the results. The objective of this study was to assess the performance of a proposed Bayesian approach to adjust for imbalances in patient level covariates when combining evidence from both types of study designs.

**Methodology/Principal Findings:**

Simulation techniques, in which the truth is known, were used to generate sets of data for randomised and non-randomised studies. Covariate imbalances between study arms were introduced in the non-randomised studies. The performance of the Bayesian hierarchical model adjusted for imbalances was assessed in terms of bias. The data were also modelled using three other Bayesian approaches for synthesising evidence from randomised and non-randomised studies. The simulations considered six scenarios aimed at assessing the sensitivity of the results to changes in the impact of the imbalances and the relative number and size of studies of each type. For all six scenarios considered, the Bayesian hierarchical model adjusted for differences within studies gave results that were unbiased and closest to the true value compared to the other models.

**Conclusions/Significance:**

Where informed health care decision making requires the synthesis of evidence from randomised and non-randomised study designs, the proposed hierarchical Bayesian method adjusted for differences in patient characteristics between study arms may facilitate the optimal use of all available evidence leading to unbiased results compared to unadjusted analyses.

## Introduction

Evidence of the effects of interventions is a critical component of health care decision making as it contributes to the comparison of alternative interventions in terms of their relative costs and effects. Such comparisons form the basis of decisions regarding the economically efficient allocation of scarce resources. An all available evidence approach to informing these decisions may require the synthesis of evidence from different types of study designs (e.g., randomised controlled trials (RCTs) and comparative non-randomised or observational studies). Recently, Bayesian hierarchical models have been proposed to combine evidence from different types of study designs such as randomised and non-randomised studies [1], [2].

Due to their inherent design, RCTs are more likely to be balanced in terms of patient characteristics between study arms than non-randomised studies, but they are subject to strict inclusion and exclusion criteria which may limit their generalisability. Despite the greater generalisability associated with non-randomised or observational studies, the increased likelihood of imbalances among the study arms compared to RCTs suggests the results may be more subject to the potential confounding effects of extraneous variables. Although other sources of bias, both internal (e.g., performance, attrition) and external (e.g., population, intervention) [3], may exist, it is the increased likelihood of imbalances among the non-randomised studies that constitutes the principal difference between randomised and non-randomised studies [4]. When these imbalances exist in factors that are also related to the outcome, bias may be introduced.

In order to address the problem of bias due to imbalances between study arms in non-randomised studies, we proposed [5] an extension to the Bayesian three-level hierarchical model, initially developed by Prevost et al. [1], and applied it to a case study. The proposed approach involved adjusting study estimates for potential imbalances using differences in patient characteristics between study arms. Results from the case study indicated a shift in the estimates for the model adjusted for differences towards the estimate for the randomised studies alone [5]. While this shift lends credence to the proposed model's ability to adjust for imbalances, these results pertain only to a single applied example.

Given the importance of using all available evidence for decision making and the increased use of Bayesian hierarchical models to combine evidence from different study types [6], [7], the objective of this paper was to assess the performance of the proposed Bayesian approach to synthesise evidence from randomised and non-randomised studies and adjust for imbalances in patient characteristics within studies. To meet the study objective, we conducted a simulation study to generate sets of randomised and non-randomised studies in which bias that could be explained by covariate imbalances was introduced in the non-randomised studies. The data were also modelled using three other Bayesian approaches: 1) results unadjusted for potential imbalances [1], 2) results adjusted for aggregate study values (e.g., mean age) [1] and 3) downweighting the potentially biased non-randomised studies [2]. The sensitivity of the results to changes in the impact of the imbalances and the relative number and size of studies of each type was also assessed.

## Methods

The following presents the four models being compared, the scenarios considered, and the methods used to conduct the simulation study.

### 2.1 Bayesian methods to combine evidence from randomised and non-randomised studies

#### 2.1.1 Unadjusted for potential imbalances (model I)

The first model presented is the Bayesian three-level hierarchical model unadjusted for potential imbalances. We undertook this analysis using a binomial model in which the odds of the event were calculated for each study and study arm level information was incorporated into the model. We assumed that for each study type (indexed by i) there were k_i_ studies (indexed by j), which allows for a different number of studies for each study type (i.e., randomised and non-randomised).

The model can be written as follows:
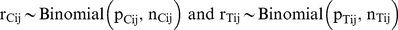
(1)

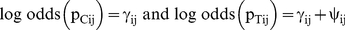
(2)

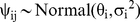
(3)

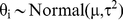
(4)(i = 1 or 2 for the 2 study types; j = 1,…,k_i_ studies).

At the first level of the model, represented by equations one and two, it was assumed that the number of events in each arm in the jth study of type i (i.e., r_Cij_ and r_Tij_ for control (C) and treatment (T), respectively) followed a binomial distribution defined by the proportion of patients who experienced the event in each arm in the jth study of type i (i.e., p_Cij_ and p_Tij_) and the total number of patients in each arm in the jth study of type i (i.e., n_Cij_ and n_Tij_). Equation two described the log odds for the event in the control (γ_ij_) and treatment (γ_ij_+ψ_ij_) arms of each of the k_i_ studies.

The second level of the model, represented by equation three, assumed that the log odds ratio comparing treatment and control, ψ_ij_, followed a normal distribution with a mean of θ_i_ (i.e., the overall intervention effect in the ith type of studies). The within-study-type variability for studies of type i was represented by σ_i_^2^. At the third level of the model, represented by equation four, the study-type effects were distributed about an overall population effect, μ, with τ^2^ representing the between-study-type variability.

Prior distributions for the unknown model parameters were intended to be vague. Normal priors with mean zero and variance 0.26 truncated to be positive, were specified for the random-effects standard deviations (σ_i_,τ). These priors support equality between studies while discounting substantial heterogeneity and represent what may be considered reasonable priors in many situations [8]. In keeping with Prevost et al. [1], Normal priors with mean zero and variance ten were used for the overall population effect (μ). Vague Normal priors with mean zero and variance 1000 were assigned to the log odds (γ_ij_'s).

#### 2.1.2 Adjustment using study arm differences (model II)

The following presents the extension of the Bayesian three-level hierarchical model (I) proposed by McCarron et al. [5]. The model was specified as before except equation three was replaced by equation five.
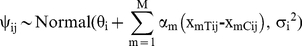
(5)(i = 1 or 2 for the 2 study types; j = 1,…,k_i_ studies; m = 1,‥,M confounders).

As shown in equation five, this model assumed that the log odds ratio, ψ_ij_, followed a normal distribution with a mean which was the sum of θ_i_ (i.e., the overall intervention effect in the ith type of studies) and a study specific bias adjustment, 
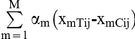
, that was proportional to the relative differences between the study arms in each of the studies. In this expression, x_mTij_ and x_mCij_ were the values of the m-th potential confounder in each of the study arms (i.e., treatment and control) in the jth study of type i while α_m_ represented the coefficient for the m-th potential confounding variable across all the studies. This variable described the impact of the imbalances on the study specific log odds ratios.

The priors for the unknown parameters were the same as for model I with the addition of a vague Normal prior with mean zero and variance 1000 for the coefficient (α_m_) for the m-th potential confounder.

#### 2.1.3 Adjustment using aggregate study values (model III)

This approach was initially proposed by Prevost et al. [1] to try to explain between study heterogeneity. The model was specified in the same way as in section 2.1.1, except equation three was replaced by equation six:
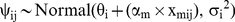
(6)(i = 1 or 2 for the 2 study types; j = 1,…,k_i_ studies; m = 1,‥,M confounders).

In this approach, x_mij_ represented the value of the m-th potential confounder aggregated across study arms (i.e., treatment and control) in the jth study of type i. This is in contrast to the previous approach which adjusted using the difference in the m-th potential confounder between the study arms. The prior distributions were the same as in the previous models.

#### 2.1.4 Downweighting using an informative prior (model IV)

The informative prior approach used by Sutton and Abrams [2] included the evidence from the non-randomised studies via the prior for the treatment effect and combined this with a likelihood based only on the data from the randomised studies.

As in Sutton and Abrams [2], we centred the informative prior for the population mean (μ) on the non-randomised pooled estimate but used a variance four times larger than that of the RCTs. Such a prior reflects scepticism regarding the non-randomised evidence and would be appropriate in situations where a researcher believes that although the non-randomised evidence provides some information, concern that serious biases may exist (e.g., as a result of imbalances in study arms) means that it should be treated with caution. The pooled estimate for the non-randomised studies was generated using a two-level Bayesian hierarchical model (simple Bayesian random-effects model). We chose to use a variance that was four times as large as that for the RCTs, because this was the variance inflation factor used by Sutton and Abrams [2]. Other choices are possible, however. The more the variance from the non-randomised studies is inflated, the more their evidence is downweighted.

### 2.2 Assessment framework

The effect of these models is to produce a weighted average of the evidence from the randomised and non-randomised studies, where the weights are determined either implicitly, as in the Bayesian three-level hierarchical models (I,II and III), or explicitly, as in the informative prior approach (model IV) [9]. The results for each of the four models were simulated under different scenarios which varied as a function of the impact of the imbalances in the non-randomised studies, and the relative number and size of studies of each type. These factors were selected as they were deemed to be the most important in terms of calculating a weighted average of the evidence from the randomised and non-randomised studies. For the purpose of this simulation study, imbalance in a single continuous covariate (i.e., age) was considered, but the analysis could be extended to adjust for other covariates [5]. Imbalances in age between study arms were only assumed for the non-randomised studies, in keeping with the general assumption that due to their design RCTs are more likely to be balanced.

Table 1 presents the parameters used in the six scenarios considered. Two different values were investigated for the impact of the imbalances in the non-randomised studies (α_m_). Log scale values of 0.10 and 0.50 were chosen as they represent lower and upper estimates of what may appear reasonable in terms of variation in the between-study log odds ratios [8]. A magnitude of 0.10 would indicate that there is not much systematic variation in the study specific log odds ratios while a magnitude of 0.50 would result in much more systematic variation. This means that, all else being equal, every one unit increase in the difference in age between study arms would result in an increase in the study specific log odds ratio of 0.10 or 0.50. For example, the impact of going from no imbalances to a one year difference in mean age between study arms would increase the study specific log odds ratio from a true value of −0.20 to values of −0.10 and 0.30 respectively.

**Table 1:** Simulation parameters for scenarios 1–6.

Criteria						
Scenario	Impact of imbalances in non-randomised studies^a^	Number of randomised studies	Number of non-randomised studies	Study arm size randomised studies^b^	Study arm size non-randomised studies^b^	True overall log odds ratio
**1**	0.10	4	4	100–500	100–500	−0.20
**2**	0.10	4	40	100–500	100–500	−0.20
**3**	0.10	4	4	100–500	500–1000	−0.20
**4**	0.50	4	4	100–500	100–500	−0.20
**5**	0.50	4	40	100–500	100–500	−0.20
**6**	0.50	4	4	100–500	500–1000	−0.20

a α_age_ measured on the log scale,

b sampled from a uniform distribution.

The impact of the precision and quantity of information contained in each of the two types of studies (i.e., randomised and non-randomised) was examined by comparing study sizes of 100 to 500 patients per arm and 500 to 1000 patients per arm for the randomised and non-randomised studies respectively and four randomised studies with 40 non-randomised studies. These values reflect the fact that non-randomised studies tend to be larger than randomised studies [2]. Also, the number of studies in a meta-analysis of RCTs in medicine tends to be small and it is common to see meta-analysis performed on five or fewer studies [10]. These values were also based on the systematic literature review comparing endovascular aneurysm repair (EVAR) and open surgical repair (OSR) [11] that informed the results of the previous case study [5]. For the six scenarios presented in Table 1, it was assumed that the true log odds ratio was −0.20, which corresponds roughly to an odds ratio of 0.82. Although this represented a much more modest treatment effect than was observed for 30-day mortality in either the randomised or non-randomised studies in the EVAR case study [5], this odds ratio may better reflect the magnitude of relative treatment effects seen in practice for other conditions.

### 2.3 Simulation study

As the truth is known, simulation studies allow one to assess model performance relative to this known truth [12]. This is in contrast to a case study, like the one in which we initially proposed model II, where the truth is not known. In order to demonstrate empirically whether model II is able to adjust for imbalances we have conducted a simulation study. The simulation study was concerned with synthesising evidence from randomised and non-randomised studies and adjusting for bias due to imbalances in the non-randomised studies, consequently we have simulated data under a model that produces imbalances in the non-randomised studies (see Figure S1).

Each simulated data set consisted of a number of hypothetical randomised (i.e., four) and non-randomised studies (i.e., four or 40) comparing treatment and control groups. The outcome was defined as a dichotomous variable indicating the occurrence or not of the event of interest (i.e., death). Each data set for each of the two study types was generated by the following model:
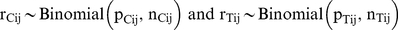
(7)

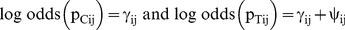
(8)

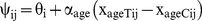
(9)The number of subjects in the control (n_Cij_) and treatment (n_Tij_) groups in the jth study of type i were assumed to be equal and were sampled from a uniform distribution of either 100 to 500 or 500 to 1000 patients. Based on the data for perioperative mortality from the previous systematic literature review [11] the probability for the event (i.e., death) in the control group (p_Cij_) in each of the k_i_ studies was drawn from a beta distribution with mean 0.04 and variance 0.001. For scenarios 1–6, the true log-odds ratio (θ_i_) was −0.20 for both the randomised and non-randomised studies. A possible explanation for the effect of treatment on mortality in our simulation study was assumed to be differences in age between study arms (x_ageTij_−x_ageCij_), as shown in equation nine. Age is related to mortality and α_age_ addresses the relationship between differences in age and mortality. The variables x_ageTij_ and x_ageCij_ are both sampled from uniform distributions based on the age distribution observed in the previous systematic literature review (i.e., x_ageTij_∼uniform(75,90), x_ageCij_∼uniform(70,85)) [11]. As randomisation will likely minimize differences between study groups, x_ageTij_ and x_ageCij_ were assumed to be equal in the randomised studies. Simulated values were generated for the number of events and subjects as well as for the age in the control and treatment groups given the impact of the imbalances (α_age_), the number of randomised and non-randomised studies, and the study size being considered.

In order to justify the number of simulations (i.e., 100), we calculated the difference in mean treatment effects for each of the models (I,III,IV) relative to the difference model (II) and compared these to the standard errors of the differences in treatment effects. This was repeated across 100 simulations for each of two seeds (starting values for the simulation). The results across both seeds suggested that 100 simulations were sufficient to average out the sampling variation. For scenario 1, for example, the differences in mean treatment effects relative to model II were 0.27 for model I, 0.28 for model III and 0.10 for model IV. The standard errors of the differences were 0.02, 0.03 and 0.02 respectively for the three comparisons. For the second seed the mean differences were 0.27, 0.28, and 0.09 respectively and the standard errors were approximately 0.02 across all three comparisons, illustrating that sampling variation was small compared to the size of the differences that were detected.

Markov chain Monte Carlo (MCMC) simulation using the Gibbs sampling technique was used to assess the models. The Brooks, Gelman & Rubin, Geweke and Heidelberger and Welch diagnostics available in the package Bayesian Output Analysis [13], performed on two chains, were used to assess convergence. To provide a sense of the convergence diagnostics we give the Brooks, Gelman & Rubin diagnostics for the overall log odds ratio (μ) for each of the models in scenario 4: the estimated values for the ratio of total variability to within-chain variability were approximately 1.01, 1, 1.01, and 1for models I through IV respectively, suggesting little between-chain variability. Based on these and the results from the other diagnostics, we decided to use a burn-in of 50 000 iterations for every model for each simulated data set except for the unadjusted and aggregate models in scenarios 2 and 5, which required a longer burn-in of 100 000 iterations to converge. After discarding the burn-in iterations, we sampled from a further 10 000 iterations with a thin rate of 20, for each of the two chains, such that summary statistics for the parameter values were based on thinned samples of 1000 iterations.

The simulated data sets were generated in R 2.9.2 [14]. The Bayesian hierarchical models (I,II,III,IV) were fitted to each generated data set in WinBUGS 1.4 [15] using the R 2.9.2 package R2WinBUGS. To validate the simulation model the mean value for α_age_ was calculated across all 100 simulations for model II and compared to the true value. The results for the six scenarios were 0.10, 0.10, 0.10, 0.52, 0.51, and 0.51 respectively. These correspond to true values of 0.10 for scenarios 1–3 and 0.50 for scenarios 4–6.

### 2.4 Criteria for assessing model performance

The median value of the overall log odds ratio (μ) was calculated for each simulated data set. The four different models for the six scenarios were then evaluated relative to the true value using the criterion of bias under repeated sampling. The estimated bias in the log odds ratio was defined as the mean value of the median log odds ratios across the simulated samples minus the true value [12]. As the results may be subject to sampling variation, we also reported the bias divided by its standard error, which is equal to the standard error of the mean of the median log odds ratios and would be expected to follow a standard normal distribution. If an estimation technique is unbiased, we would expect the observed bias divided by its standard error (Z-statistic) to lie between −1.96 and +1.96 ninety-five percent of the time. Formulas for the various calculations are given in Figure S1.

## Results

Table 2 shows the point estimates for the mean of the median log odds ratios, and the associated standard errors of the mean median log odds ratios as well as the estimated bias and Z-statistics for each of the four models in the six scenarios. As shown in this table, the estimates of the pooled effect size appear to be unbiased for the model adjusted for differences (model II) across all six scenarios. The informative prior approach appears to give less biased results than the model adjusted for aggregate study values while bias is roughly equal for both the model adjusted for aggregate values and the unadjusted model. An increase in the study arm size for the non-randomised studies relative to the randomised studies tends to increase the precision of the estimates for all of the models. However, combining evidence from four randomised studies and 40 non-randomised studies seems to increase the precision of the estimates the most compared to the other scenarios. In general, as might be expected, there is more variability in the model estimates when the assumed value of the impact of imbalances in age across all of the studies (α_age_) is greater. The most extreme cases of bias appear to occur with the aggregate and unadjusted models in scenario five, when the value of α_age_ is 0.50 and there are four randomised and 40 non-randomised studies, and scenario two, when α_age_ is 0.10 and there are 40 non-randomised studies. However, as shown in table 2, the extent of the bias is more pronounced in scenario five compared to scenario two, where the magnitude of the impact of the imbalances is relatively smaller.

**Table 2:** Simulation results comparing Bayesian hierarchical models for scenarios 1–6.

Scenario	Model	Mean median log odds ratio	Standard error mean median log odds ratio	Bias	Z-statistic
**1**	Unadjusted (I)	0.06253	0.02268	0.26253	11.57665
	Adjusted for differences (II)	−0.20836	0.02374	−0.00836	−0.35207
	Adjusted for aggregate values (III)	0.07407	0.02622	0.27407	10.45383
	Informative prior (IV)	−0.11156	0.02437	0.08844	3.62828
**2**	Unadjusted (I)	0.18750	0.01330	0.38750	29.12960
	Adjusted for differences (II)	−0.20216	0.01010	−0.00216	−0.21398
	Adjusted for aggregate values (III)	0.19520	0.01355	0.39520	29.17138
	Informative prior (IV)	−0.12240	0.02385	0.07760	3.25356
**3**	Unadjusted (I)	0.05473	0.02079	0.25473	12.25142
	Adjusted for differences (II)	−0.23125	0.01816	−0.03125	−1.72104
	Adjusted for aggregate values (III)	0.05979	0.02189	0.25979	11.86562
	Informative prior (IV)	−0.13908	0.02235	0.06092	2.72561
**4**	Unadjusted (I)	0.87357	0.06602	1.07357	16.26034
	Adjusted for differences (II)	−0.22000	0.02535	−0.02000	−0.78904
	Adjusted for aggregate values (III)	0.98388	0.07572	1.18388	15.63405
	Informative prior (IV)	0.85343	0.09327	1.05343	11.29504
**5**	Unadjusted (I)	1.14790	0.03313	1.34790	40.67943
	Adjusted for differences (II)	−0.20083	0.01146	−0.00083	−0.07268
	Adjusted for aggregate values (III)	1.28827	0.03734	1.48827	39.85580
	Informative prior (IV)	0.64133	0.05340	0.84133	15.75488
**6**	Unadjusted (I)	0.70170	0.06319	0.90170	14.27030
	Adjusted for differences (II)	−0.19981	0.01721	0.00019	0.01117
	Adjusted for aggregate values (III)	0.78753	0.06303	0.98753	15.66646
	Informative prior (IV)	0.69489	0.09509	0.89489	9.41122

**Figure 1:**
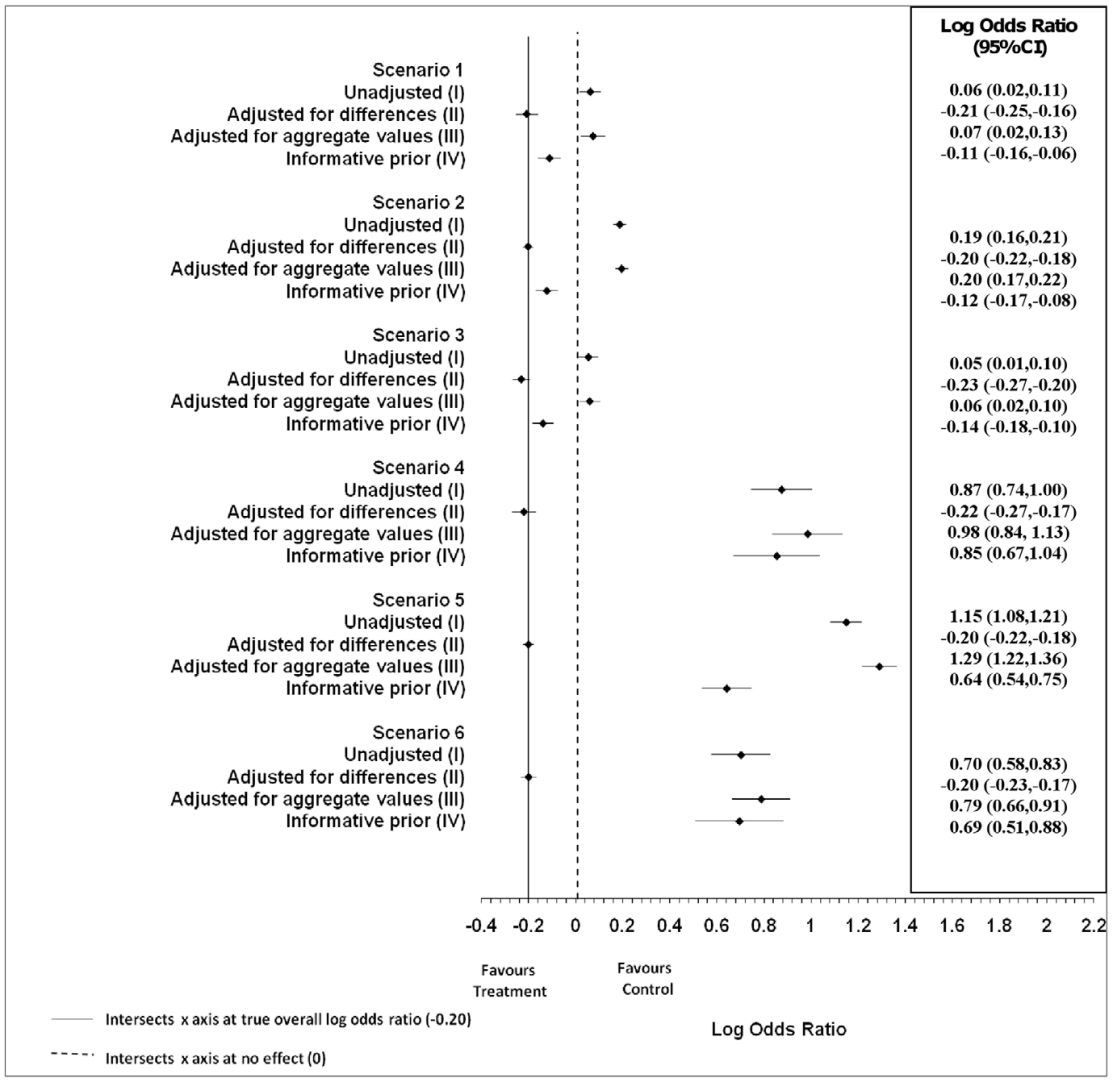
Overall log odds ratios for Bayesian hierarchical models scenarios 1–6. The overall log odds ratios (μ) and associated 95% confidence intervals (CIs) from the simulations are presented for scenarios 1–6. A solid line intersects the x axis at the true overall log odds ratio (i.e., −0.20). A dashed line intersects the x axis at no effect (i.e., 0).

Figure 1 presents the point estimates and the confidence intervals for the overall log odds ratio (μ) for each of the models in the six scenarios. Comparing the point estimates to a log odds ratio of zero (i.e., no effect) indicates that among the aggregate and unadjusted models and even the informative prior, for scenarios 4–6, the impact of the imbalances is such that it alters the estimate as to whether or not the treatment is effective, thus deviating from the truth.

## Discussion

This simulation study demonstrated that when bias in the non-randomised studies can be explained by covariate imbalances between study arms, the proposed Bayesian three-level hierarchical model adjusted for differences in patient characteristics within studies can handle this problem. Using simulation techniques, wherein the truth is known, we have been able to produce empirical evidence that this is the case. Failure to take into account these imbalances could bias the results.

Specifically, six scenarios incorporating different aspects of the impact of the imbalances and the relative numbers and sizes of each study type were considered. The results from the model adjusted for differences in patient characteristics within studies were, in every scenario, unbiased and closest to the true value compared to the results from the other models. This trend was robust to changes in the magnitude of the impact of the imbalances across studies as well as to both the relative number and size of studies being combined. Results also showed that none of the previously proposed Bayesian approaches could handle the issue of bias due to covariate imbalances. In certain instances, the bias observed among the other models was such that it changed the treatment estimate from one of benefit to one of harm. This could have implications in terms of health care decision making.

A practical limitation of the study concerns the number of simulations. There are no exact standards for the number of simulations necessary to average out sampling variation. We had initially considered performing 1000 simulations, but given the breadth of the study in terms of the number of scenarios considered and the associated run times, which ranged from five to 40 hours (1.83 GHz processor) for the 100 simulations, depending on the scenario, we determined that this would not be feasible. All of the parameters in each of the models were sampled and none were marginalised. This was done to ensure that the appropriate probabilistic dependence between the unknown parameters was propagated through the model. This could be particularly important when propagating inferences which are likely to be strongly correlated. For example, the current study considers both baseline levels and treatment differences estimated from the same studies [8]. In addition, the study is based on the assumption that there is also some association with imbalances in patient characteristics. As such it was important to sample all parameters in our simulation study. In other cases, perhaps, some parameters could be marginalised which could potentially improve the speed of the algorithm. Concerns regarding the number of simulations conducted were mitigated by comparing the effect sizes relative to the standard errors for each of two data sets. The results of these comparisons suggested we could be reasonably confident that the number of simulations was adequate.

Another potential limitation is that we assumed that the only source of variation between study estimates was due to imbalances between treatment arms in a single patient characteristic. As such, the underlying study type effects in both the randomised and non-randomised studies were assumed to be the same, which may not always be true. In practice, there may be other unexplained reasons why the estimates may differ. For example, patients enrolled in RCTs may be comparatively younger than those enrolled in non-randomised studies. The result of incorporating different values for the study type treatment effects is that there is no longer one true underlying effect, as there was in the six scenarios we considered. As the objective of the simulation study was to evaluate the performance of the proposed model in terms of adjusting for bias due to covariate imbalances, we did not address this issue in our study. Such an analysis would likely require a separate simulation study in which each scenario considered would involve its own base case assuming no imbalances. This would allow one to distinguish between the borrowing of strength across study types that is part of Bayesian hierarchical modelling and the appropriate adjustment for imbalances. This is left for future research. Future research could also assess the practical implications of these results within a decision analytic model. Another potential area of research could be the choice of prior distribution for the random-effects standard deviations (σ_i_,τ). In contrast to the half-normal priors used in the current analysis, other suggestions include an inverse gamma distribution such as 1/σ_i_^2^∼Gamma[0.001,0.001]. Though, because such a distribution gives a high weight near zero for the standard deviation, the true variability may be underestimated [8], [10]. As the current analysis relies on the existence of within-study-type and between-study-type variability, such a prior could be problematic, especially in those scenarios with only four non-randomised studies.

Despite potential limitations, we believe the results of this simulation study demonstrate the ability of the Bayesian three-level hierarchical model adjusted for differences to account for imbalances in patient characteristics within non-randomised studies that could bias the results. Such an approach does, however, rely on authors reporting the main characteristics of their study populations. This is important as the unadjusted model performed poorly in the presence of imbalances between study arms, as shown in our simulations. Unfortunately, few studies report all relevant covariates [4]. For example, in the initial case study, over half of the non-randomised studies were missing information on at least one covariate. Based on the results of our study, and the performance of the proposed approach, authors should be encouraged to improve the reporting of covariate information as this would facilitate adjustment for future evidence synthesis. The performance of the informative prior approach depends on how well one anticipates the impact of the imbalances on the results and downweights the evidence accordingly. Though the factor we used to inflate the variance and downweight the non-randomised studies was based on Sutton and Abrams [2], this value was somewhat arbitrary. In practice the selection of an appropriate discount factor would require a careful consideration of the relative weight and information each study type should contribute to the analysis. Nonetheless, the factor of four used for model IV in the current study means that in calculating a weighted average of both study types, the randomised studies would contribute the majority of the information. This reflects the existence of scepticism regarding the evidence generated by the non-randomised studies, but assumes there is still some value in combining these studies with the randomised studies. As has been demonstrated, by holding constant the amount by which the non-randomised studies were downweighted, downweighting is not an automatic procedure, nor does it explicitly address the potential for imbalances in patient characteristics within individual studies. Only one of the methods for downweighting used in the case study was considered in this analysis. The number of failures that occurred when simulating values for the prior constraint method [1] suggested that it could not be used reliably in the situations being investigated. However, the results of the case study suggest it is unlikely that this method would be able to handle the covariate imbalances, especially in those scenarios where the relative number or size of the non-randomised studies was greater compared to the randomised studies. Adjustment using aggregate study values attempts to explain heterogeneity across studies by adjusting for variation in study level characteristics. However, the absence of variation in mean age across studies does not preclude the presence of imbalances in age within studies. This will not be adjusted for using aggregate study values.

Based on the six scenarios considered, covariate adjustment using differences in patient characteristics between study arms (i.e., model II) provides a way of adjusting for imbalances that is robust to changes in the magnitude of the impact of the imbalances and the relative number and size of the studies of each type (i.e., randomised or non-randomised studies). This is important as this new methodology provides a way to synthesise randomised and non-randomised studies by adjusting for bias in non-randomised studies that is due to imbalances between treatment arms. Where informed health care decision making requires the synthesis of evidence from randomised and non-randomised study designs, such Bayesian hierarchical models adjusting for covariate imbalances could facilitate the optimal use of all available evidence.

## Supporting Information

Figure S1 **Flow chart depicting data simulation, analysis and output for scenarios 1–6.** The flow chart depicts the simulation of the data in R, the analysis of the simulated data in WinBUGS and the statistics used to assess the performance of the four models.
(PDF)
