# Supplementary material for: Bayesian Hierarchical Models Combining Different Study Types and Adjusting for Covariate Imbalances: A Simulation Study to Assess Model Performance
Source: PLoS One. 2011 Oct 10;6(10):e25635. doi: 10.1371/journal.pone.0025635 (PMC3189931; doi:10.1371/journal.pone.0025635)
Supplement: Figure S1 — Flow chart depicting data simulation, analysis and output for scenarios 1–6. The flow chart depicts the simulation of the data in R, the analysis of the simulated data in WinBUGS and the statistics used to assess the performance of the four models. (PDF) [file pone.0025635.s001.pdf]

DATA SIMULATION: R

S = 100	Number of simulations
θ = -0.20	True log odds ratio
α_{age} = 0.10	Impact of imbalances in age in scenarios 1,2, and 3
α_{age} = 0.50	Impact of imbalances in age in scenarios 4,5, and 6

Non-randomised Studies

N = 4	Number of non-randomised studies for scenarios 1,3,4, and 6
N = 40	Number of non-randomised studies for scenarios 2 and 5
nC ~ uniform(100,500)	Control group study arm size for scenarios 1,2,4, and 5
nC ~ uniform(500,1000)	Control group study arm size for scenarios 3 and 6
nT = nC	Treatment group study arm size
pC ~ beta(1,25)	Control group event probability
rC ~ binomial(nC,pC)	Control group number of events
λ = log(pC/(1 – pC))	Control group log odds of an event
ageC ~ uniform(70,85)	Control group mean age
ageT ~ uniform(75,90)	Treatment group mean age
$\beta = \lambda + \theta + \alpha_{age}$ *(ageT – ageC)	Treatment group log odds of an event
pT = exp(β) / (1 + exp(β))	Treatment group event probability
rT ~ binomial(nT,pT)	Treatment group number of events
age = (ageC + ageT) / 2	Study mean age

Randomised Studies

Nr = 4	Number of randomised studies
nCr ~ uniform(100,500)	Control group study arm size
nT r= nCr	Treatment group study arm size
pCr ~ beta(1,25)	Control group event probability
rCr ~ binomial(nC,pC)	Control group number of events
λr = log(pCr/(1 – pCr))	Control group log odds of an event
ageCr ~ uniform(70,90)	Control group mean age
ageTr = ageCr	Treatment group mean age
βr= λr + θ + α_{age} *(ageTr – ageCr)	Treatment group log odds of an event
pTr = exp(βr) / (1 + exp(βr))	Treatment group event probability
rTr ~ binomial(nTr,pTr)	Treatment group number of events
ager = (ageCr + ageTr) / 2	Study mean age

ANALYSIS: WinBUGS

N = 4	Number of non-randomised studies for scenarios 1,3,4, and 6
N = 40	Number of non-randomised studies for scenarios 2 and 5
Nr = 4	Number of randomised studies
i = 1,.......,N	For non-randomised studies
j = 1,.......,Nr	For randomised studies
nCi	Control group study arm size non-randomised study i
nTi	Treatment group study arm size non-randomised study i
rCi	Control group number of events non-randomised study i
rTi	Treatment group number of events non-randomised study i
nCrj	Control group study arm size randomised study j
nTrj	Treatment group study arm size randomised study j
rCrj	Control group number of events randomised study j
rTrj	Treatment group number of events randomised study j
ageCi	Control group mean age non-randomised study i
ageTi	Treatment group mean age non-randomised study i
ageCrj	Control group mean age randomised study j
ageTrj	Treatment group mean age randomised study j
agei	Study mean age non-randomised study i
agerj	Study mean age randomised study j

OUTPUT

θ_k = median log odds ratio for simulation k

θ_{bar} = Mean median log odds ratio = $\sum_{k=1}^{S} \theta_k / S$

SD = Standard deviation = $\sqrt{[1/(S-1)]\sum_{k=1}^{S}(\theta_k - \theta_{bar})^2}$

SE = Standard error mean median log odds ratio = $SD/\sqrt{S}$

Bias = $\theta_{bar} - \theta$

Z-statistic = Bias / SE
